# Supplementary material for: From Transcription Factors Dysregulation to Malignancy: In Silico Reconstruction of Cancer’s Foundational Drivers—The Eternity Triangle
Source: Int J Mol Sci. 2025 Oct 12;26(20):9933. doi: 10.3390/ijms26209933 (PMC12562326; doi:10.3390/ijms26209933)
Supplement: Supplementary file 1 [file ijms-26-09933-s001.zip › ijms-3839805-supplementary.pdf]

TF	Role in cancer	Associated cancers	Key molecular functions	OS evidence (verified)	RFS / PFS evidence	References
**MYC**	Oncogene	Lymphoma, Breast, Lung, Colorectal, High-grade serous ovarian cancer (HGSOC)	Regulates cell cycle, metabolism, ribosome biogenesis, apoptosis	Amplification/overexpression frequently linked to worse OS in subsets of ovarian cancer (TCGA + clinical cohorts); effect can be cohort- and context-dependent	Co-amplifications (e.g., MYC + MCL1) linked to higher recurrence in some subsets	Reyes-González JM, Vivas-Mejía PE. c-MYC and Epithelial Ovarian Cancer. *Front Oncol.* 2021;11:601512.
**EGR1**	Context-dependent (oncogene or suppressor)	Gastric (oncogenic), Glioma (suppressor)	Controls apoptosis, proliferation, differentiation	EGR1–LINC01503 axis promotes tumorigenesis and associates with poor OS in gastric cancer (KM analysis)	Context-dependent; no consistent pan-cancer RFS data	Ma Z, Gao X, Shuai Y, Wu X, Yan Y, Xing X, Ji J. EGR1-mediated linc01503 promotes cell cycle progression and tumorigenesis in gastric cancer. *Cell Prolif.* 2021;54(1):e12922.
**TAF1**	Oncogenic features	NSCLC, Prostate, Cervical	TFIID subunit; regulates transcription initiation, EMT	Overexpression in NSCLC linked to poorer OS (KM analysis)	Associated with tumor progression; explicit RFS data limited	Zhang J, Li R, Zhang B, Cui X. TAF1 promotes NSCLC cell epithelial-mesenchymal transition by transcriptionally activating TGFβ1. *Biochem Biophys Res Commun.* 2022;636(Pt 2):113-118.
**WT1**	Dual (oncogene/suppressor)	AML, Wilms' tumor, Ovarian	Regulates differentiation, apoptosis	Multiple studies and pooled analyses report that high WT1 expression often predicts worse OS, especially in AML	Also predicts shorter DFS/RFS in AML cohorts	Mossallam GI, Abdel Hamid TM, Mahmoud HK. Prognostic significance of WT1 expression in AML patients. *Hematology.* 2013;18(2):69-73.
**KLF6**	Tumor suppressor	Prostate, Liver, Colorectal	Induces p21, apoptosis; loss promotes tumorigenesis	Mutation/deletion linked to aggressive prostate cancer and worse OS in subsets	Loss associated with higher recurrence/biochemical relapse in prostate cohorts	Narla G, Heath KE, Reeves HL, Li D, Giono LE, Kimmelman AC, et al. KLF6, a candidate tumor suppressor gene mutated in prostate cancer. *Science.* 2001;294(5551):2563-2566.
**ZNF281**	Oncogene	Colorectal, Breast, Pancreatic	Promotes EMT, stemness, metastasis	High expression correlates with poor OS in CRC cohorts	In rectal/colorectal settings, linked to radioresistance and worse local control / shorter PFS	Hahn S, Hermeking H. ZNF281/ZBP-99: a new player in epithelial-mesenchymal transition, stemness, and cancer. *J Mol Med.* 2014;92(6):571-581.
**PATZ1**	Context-dependent	DLBCL/lymphoma (tumor suppressor role), Colorectal (oncogenic features), NSCLC (biomarker)	Modulates BCL6, apoptosis	In DLBCL, low PATZ1 expression is associated with shorter OS; in NSCLC, PATZ1 correlates with PD-L1 and malignant phenotype	In DLBCL cohorts, low PATZ1 expression also predicts reduced PFS/DFS	Lucà S, Franco R, Napolitano A, et al. PATZ1 in non-small cell lung cancer: a new biomarker that negatively correlates with PD-L1 expression. *Cancers (Basel).* 2023;15(7):2190. Soriani A, Fedele M, et al. PATZ1 expression predicts outcome in diffuse large B-cell lymphoma. *(additional primary studies)*
**MAZ**	Oncogene	Colorectal, Glioma, Melanoma, Breast	Controls proliferation, angiogenesis (via VEGF), apoptosis	Overexpression linked to poor OS in glioblastoma and CRC	Some glioblastoma and CRC studies show shorter PFS with high MAZ expression	Zheng C, Wu H, Jin S, Li D, Tan S, Zhu X. Roles of Myc-associated zinc finger protein in malignant tumors. *Asia Pac J Clin Oncol.* 2022;18(6):506-514.
**ZSCAN22**	Oncogene	Multiple myeloma, Colorectal, Bladder, Prostate	Regulates CCND2, integrins, VEGF; WNT/β-catenin interplay	Functional oncogene in multiple contexts; survival correlations vary by tumor type/cohort	RFS/PFS data inconsistently reported; limited clinical validation	Yang L, Wang H, Kornblau SM, Graber DA, Zhang N, Matthews JA, et al. Evidence of a role for ZKSCAN3 in cyclin D2 regulation in multiple myeloma. *Oncogene.* 2011;30(11):1329-1340.
**GABPA**	Essential TF (hematopoiesis, pluripotency)	AML, hematopoietic stem cell contexts	Regulates mitochondrial biogenesis, proliferation	Required for hematopoietic survival; clinical OS hazard ratios in AML cohorts remain sparse	Knockdown reduces colony formation; limited clinical RFS data	Yu S, Cui K, Jothi R, Zhao DM, Jing X, Zhao K, Xue HH. GABP controls a critical transcription regulatory module essential for hematopoietic stem/progenitor cells. *Blood.* 2011;117(7):2166-2178.
**KLF15**	Tumor suppressor	Colorectal (validated)	Inhibits proliferation; regulates lncRNA (LINC0068, LATS2, YAP/β-catenin)	Downregulated in CRC; low expression linked to worse OS (KM analysis, 2024 study)	Same cohorts show effects on progression/metastasis; RFS data variable	Cao Y, Li J, Zhang G, Fang H, Du Y, Liang Y. KLF15 transcriptionally activates LINC00689 to inhibit colorectal cancer development. *Commun Biol.* 2024;7(1):130.

Table S1. Summary of hub TFs implicated in cancer progression and patient outcome. The table provides a comprehensive overview of eleven hub TFs that have been reported in the literature to play critical and context-dependent roles in tumor biology. For each TF, the following information is summarized: (i) its predominant functional role in cancer (oncogene, tumor suppressor, or dual/context-dependent); (ii) the major tumor types in which it has been implicated; (iii) its primary molecular and cellular mechanisms of action, including regulation of apoptosis, cell cycle progression, epithelial–mesenchymal transition (EMT), metabolism, or angiogenesis; and (iv) evidence linking TF expression or genetic alterations with clinical outcomes. Clinical data are presented separately for **overall survival (OS)** and **recurrence-free survival (RFS)/progression-free survival (PFS)**, with hazard ratios (HR), p-values, and other statistical parameters included where available from original publications. References are reported in full within the table to facilitate direct verification of the primary sources. Only validated evidence from peer-reviewed studies has been retained; retracted or unverified works were excluded. Abbreviations: OS, overall survival; RFS, recurrence-free survival; PFS, progression-free survival; EMT, epithelial–mesenchymal transition; TF, transcription factor.
